# Perceived need for HIV testing among rural and urban African American cocaine users

**DOI:** 10.1186/1940-0640-10-S1-A74

**Published:** 2015-02-20

**Authors:** Patricia B Wright, Tyrone F Borders, Brenda M Booth, Xiaotong Han, Katharine E Stewart

**Affiliations:** 1 College of Nursing, University of Arkansas for Medical Sciences, Little Rock, AR, 72205, USA; 2 Department of Health Services Management, College of Public Health, University of Kentucky, Lexington, KY, 40536, USA; 3 Division of Health Services Research, Psychiatric Research Institute, University of Arkansas for Medical Sciences, Little Rock, AK, 72205, USA; 4 General Administration, University of North Carolina – Charlotte, Charlotte, NC, 28223, USA

## Background

Incidence rates of HIV continue to be disproportionately high among African Americans compared to other races, especially in the South. Although we have some information about testing among African Americans, most surveys do not include homeless, impoverished, or other disenfranchised and therefore hard-to-reach groups, many of whom may be particularly high-risk for HIV infection, and few studies have compared HIV testing among rural and urban African Americans. The purpose of this study, therefore, is to address this gap by systematically examining perceived need for HIV testing among rural as compared to urban African American cocaine users.

## Methods

This is a cross-sectional, community-based study of 400 not-in-treatment African American cocaine users residing in selected rural and urban areas of Arkansas. We used respondent-driven sampling methods to fill our sample. Face-to-face computer-assisted interviews were conducted in study offices lasting 1½ – 2 hours. A logistic regression model was used to examine the association between perceived need for HIV testing and rural/urban residence, while controlling for demographic and clinical characteristics. Covariates in the model included: demographics (age, gender, education, health insurance), clinical characteristics (lifetime drug abuse treatment, lifetime mental health treatment, routine health-care visit in past year, general health status, ever being tested for hepatitis C or gonorrhea, number of times tested for HIV), access factors (ease of access to HIV testing, availability of HIV testing, skepticism towards medical care, stigma for HIV testing) and behavioral risk factors (perceived risk of acquiring HIV, number of sex partners, inconsistent condom use, ever trading sex for foods or drugs).

## Results

We did not find significant differences among rural and urban African American cocaine users’ perceived need for HIV testing. Female gender, perceived risk for acquiring HIV, stigma, and receipt of testing for other sexually transmitted diseases were each positively and significantly related to perceived need to be tested for HIV in multivariate regression analysis in which all other variables were held constant. Receipt of routine medical care in the past year, being tested for HIV from 3–5 times, and skepticism towards medical care were negatively and significantly related to perceived need for HIV testing. Behavioral risk factors or general health status were not significantly associated with perceived need for HIV testing. Access to testing was not perceived as a significant barrier to testing.

## Conclusions

All participants were not-in-treatment, non-injecting drug users, and most reported engaging in risky sex behaviors; however, risky behavior was not associated with perceived need to be tested for HIV. This research underscores the need to reframe HIV prevention programs targeting African American substance users to separate the importance of testing from risky behaviors and to frame testing as taking care of oneself and one’s health.

